# CU104, a novel barrier function enhancer, improves colitis via modulation of barrier function and immune cell recruitment

**DOI:** 10.3389/fimmu.2026.1767762

**Published:** 2026-03-10

**Authors:** I Seul Park, Ji Hyung Kim, Dongyeop Kim, Ye Won Kim, Yoojin Shin, Ki Beom Kim, Haiying Zhang, Tae Il Kim, Seung Won Kim, Young-Guen Kwon, Jae Hee Cheon

**Affiliations:** 1 Department of Internal Medicine and Institute of Gastroenterology, Graduate School of Medical Science, Brain Korea 21 Project, Yonsei University College of Medicine, Seoul, Republic of Korea; 2 Department of Biochemistry, College of Life Science and Biotechnology, Yonsei University, Seoul, Republic of Korea; 3 Curacle Co. Ltd, Seoul, Republic of Korea; 4 Severance Biomedical Science Institute, Yonsei University College of Medicine, Seoul, Republic of Korea

**Keywords:** actin dynamics, barrier function, immune cell recruitment, interferon regulatory factor, interleukin 10

## Abstract

**Background:**

Inflammatory bowel disease (IBD), including Crohn’s disease and ulcerative colitis, is a chronic and relapsing condition with complex pathogenesis and limited therapeutic options. The efficacy of CU104, a novel blocker of endothelial dysfunction, in IBD models is poorly understood. Moreover, its precise cellular or molecular mechanisms in colitis remain unknown.

**Methods:**

To evaluate the therapeutic potential of CU104, we tested CU104 in two colitis models: dinitrobenzene sulfonic acid (DNBS)–induced colitis in wild-type mice and dextran sodium sulfate (DSS)–challenged colitis in IL-10 knockout mice. Additionally, we used Caco-2, HCT-116, and HT-29 cells to assess CU104 effects on intestinal barrier function (FITC-dextran permeability and TEER), inflammatory signaling (reporter assays), actin dynamics, and gene expression (gene expression profiling and immune assays).

**Results:**

CU104 demonstrated potent suppressive effects on innate immune responses, intestinal and vascular barrier dysfunctions, and immune cell recruitment in these colitis models. Furthermore, CU104 inhibited the activation of the transcription factors nuclear factor kappa-light-chain-enhancer of activated B cells and interferon regulatory factor, as well as the ezrin/radixin/moesin (ERM) signaling pathway, both *in vitro* and *in vivo*, by modulating actin dynamics. Consistent with these findings, CU104 improved the functions of vascular and intestinal barriers and regulated immune cell recruitment during inflammation.

**Conclusions:**

Collectively, our findings demonstrate that CU104 can regulate actin dynamics and inflammatory signaling pathways, highlighting potential therapeutic targets for IBD.

## Introduction

Inflammatory bowel disease (IBD), including Crohn’s disease (CD) and ulcerative colitis (UC), is a chronic and recurring immune-mediated condition (1). Genetic variants in over 240 genes and the resulting aberrant immune responses have been identified as key factors in the pathogenesis of IBD. Barrier function defects and immune dysregulation can lead to increased exposure to pathogens and abnormal immune responses, resulting in elevated levels of inflammatory cytokines. These cytokines exacerbate the permeability of intestinal epithelial cells (IECs) and blood vessels, facilitating immune cell infiltration and recruitment, thus boosting the immune responses (2). IBD medications, such as 5-aminosalicylic acid (5-ASA), corticosteroids, immunosuppressants, and advanced therapies, including biologics and small molecules, primarily target immune modulation (3). However, these drugs often have limited efficacy and adverse effects (4). Consequently, recent efforts have focused on exploring novel therapeutic targets, necessitating a deeper understanding of the complex nature of IBD pathogenesis (5).

CU104, formerly known as Sac-1004 or CU06-1004 (6), is an orally active, lipophilic small-molecule compound that has been reported to enhance endothelial cell survival under serum-deprivation conditions and to preserve endothelial barrier integrity against permeability-inducing stimuli such as VEGF, histamine, thrombin, and IL-1β *in vitro* (6–9). In addition, CU06–1004 has been reported to suppress endothelial inflammatory activation, including reduced ICAM-1 and VCAM-1 expression via inhibition of NF-κB signaling, and to mitigate vascular leakage and inflammation across multiple *in vivo* disease models (6, 8, 9). We recently reported that CU104 improves vascular integrity and the expression of tight junction proteins in endothelial cells and inflammatory adhesion molecules in a dextran sodium sulfate (DSS)-induced colitis model (10), which resembles UC (11). However, the mechanisms by which CU104 attenuates intestinal barrier disruption and affects immune cells, as well as the specific cellular or molecular targets in colitis, remain elusive. Furthermore, its therapeutic efficacy has not yet been evaluated in other IBD models, including CD-like models. To address this gap, we employed hapten-induced dinitrobenzene sulfonic acid (DNBS) and dextran sodium sulfate (DSS)-induced colitis models commonly used in IBD studies. This DNBS model exhibits inflammatory features resembling those of CD, but distinct from those of the DSS model, as previously reported (10, 12). The interaction between proteins and hapten molecules induced by DNBS leads to intestinal barrier damage, triggering both innate and adaptive immune responses (13). Although these models predominantly represent acute-to-subacute inflammatory phases, they reliably recapitulate the pathophysiological changes seen during clinical exacerbations of IBD.

Interleukin 10 (IL-10) is a multifunctional cytokine that regulates various immune cells and cytokines (14). IL-10 knockout (IL-10 KO) mice, a well-known genetic model of IBD, spontaneously develop chronic colitis, highlighting the critical role of IL-10 in immune homeostasis. IL-10 deficiency disrupts the balance between CD4^+^ T cells and neutrophil homeostasis, contributing to the development of colitis (15). This deficiency also exhibits a CD-like phenotype characterized by Th1-like inflammation, similar to that observed in the DNBS-induced colitis (16).

In this study, we investigated the molecular mechanisms underlying the anticolitic effects of CU104, with a focus on its impact on intestinal epithelial barrier function, immune modulation, and immune cell recruitment using two acute DNBS-induced colitis and DSS-induced IL-10 KO models. We also explored the regulation of IL-10-independent innate immune responses, particularly through signaling pathways involving nuclear factor kappa-light-chain-enhancer of activated B cells (NF-κB) and interferon regulatory factors (IRF). Finally, we investigated the impact of CU104 on the ezrin/radixin/moesin (ERM) signaling pathway, which regulates actin dynamics and influences intestinal barrier function and immune cell recruitment. By comprehensively examining these aspects, we aimed to provide insights into the therapeutic potential of CU104 and its specific targets in IBD.

## Methods

### DNBS-induced mouse colitis model

Eight-week-old male BALB/c mice were purchased from Daehan Biolink (Eumsung, Korea). All animals were acclimated for a week under controlled conditions (22 °C ± 1 °C) and 50%–60% humidity, with a 12-h light/12-h dark cycle. Mice had free access to purified water and standard chow and were maintained under specific pathogen-free conditions at Yonsei University. Mice were fasted overnight prior to DNBS administration. On day 0, distal colitis was induced by administering 2 mg of DNBS in 50% ethanol (100 µl). Mice were maintained in an upright position for 30 seconds to ensure that the solution remained in the colon. The control group received 50% ethanol alone. A water-gavaged mouse (Water group) was used as the control. Vehicle (olive oil; used as the vehicle control because CU104 and comparator drugs were dissolved in olive oil for oral administration), CU104 (10 mg/kg), tofacitinib (30 mg/kg) (17), or ozanimod (1 mg/kg) (18) were administered daily (q.d.) by oral gavage from Day 0 until the day before sacrifice. The doses of CU104 (10 and 20 mg/kg) were selected based on prior preclinical studies (6, 8, 10). Starting from Day 0 until Day 8, the Water and DSS+Veh groups were orally administered the vehicle daily. Similarly, the DSS+CU104 (10 or 20 mg/kg) and DSS + 5-ASA (100 mg/kg) groups received their respective treatments daily via oral gavage throughout the entire period prior to sacrifice on Day 9. Ozanimod, a sphingosine-1-phosphate receptor (S1PR) modulator that modulates lymphocyte trafficking, was included as a comparator control (18, 19). Body weight, fecal blood, and stool consistency were recorded daily. The disease activity index (DAI) score was determined using the following criteria: (1) Weight loss was categorized as 0 (< 1%), 1 (1%–5%), 2 (5%–10%), 3 (10%–15%), or 4 (>15%); (2) Stool consistency was rated as 0 (normal), 2 (loose stool), or 4 (diarrhea); and (3) fecal blood was evaluated as 0 (no blood), 2 (visible red blood), or 4 (black or tarry stool indicating severe bleeding).

### Induction of DSS-induced colitis in IL-10 KO mice

Male and female IL-10 KO mice were purchased from the Jackson Laboratory (Bar Harbor, Maine, USA) and acclimated for a week before the experiment. Nine-week-old male IL-10 KO mice were randomly assigned to five groups: Water, DSS+Veh (vehicle), DSS+CU104 (10 mg/kg), DSS+CU104 (20 mg/kg), and DSS + 5-ASA (100 mg/kg) group. The Water group was given sterile tap water, whereas the other groups were administered a 2% (w/v) solution of DSS (m.w. 36,000-50,000; MP Biomedicals, Irvine, CA, USA) for 7 days, followed by sterile tap water for two days. Starting from Day 0 to Day 8, the Water and DSS+Veh groups were orally administered the vehicle daily. The DSS+CU104 (10 mg/kg) and DSS+CU104 (20 mg/kg) groups received oral administration of CU104 (dissolved in olive oil), while the DSS + 5-ASA group received 5-ASA (Sigma, PHR1060, dissolved in olive oil at 100 mg/kg) until Day 8. On day 9, mice were euthanized using CO_2_ gas (Hana Pharm, Seoul, Korea). Peritoneal cells were collected for M1/M2 macrophage analysis, and the colon and spleen were excised for the further analysis. The colon length was measured from the cecum to the anus. A 1 cm segment from the anus was collected for histopathological evaluation. From the remaining colon tissue, a 0.5 cm section representing the third 3/4 of the length was processed for RNA analysis, while the remaining segments were frozen for further analysis, as previously described (20).

### Histopathological assessment

Colon tissue samples were fixed overnight in 10% neutral buffered formalin solution and paraffin-embedded. Paraffin blocks were sectioned at a thickness of 4 μm and finally stained with hematoxylin and eosin (H&E), Alcian Blue, or periodic acid-Schiff (PAS) for histological evaluation and scoring as previously described (10). Images of the stained tissues were captured using a light microscope (Olympus BX41; Olympus Optical, Tokyo, Japan). The severity of symptoms such as crypt damage and inflammatory cell infiltration was assessed using a scoring system. Histological damage was scored (0–6) by summing two parameters: inflammatory infiltration (1: mucosa; 2: submucosa; 3: transmural) and intestinal architecture (1: focal erosions; 2: erosions/ulceration; 3: extended ulceration/pseudopolyps). All images were anonymized, and histological scoring was performed in a blinded manner to minimize observer bias. Additionally, goblet cell loss in colon tissues was quantified using ImageJ software (version 1.53e; National Institutes of Health, Bethesda, MD, USA).

### Flow cytometric analysis

The populations of Th17 and Treg cells, as well as M1/M2 macrophages, were analyzed using splenocytes and peritoneal cells, following the previously described methods. Cell suspensions were prepared in Dulbecco’s phosphate-buffered saline (DPBS) solutions containing calcium chloride (DPBS) and 2% FBS. Cells (1 × 10^6^) were blocked with normal mouse and rat serum (Thermo Fisher Scientific, San Jose, CA, USA) and then incubated with appropriate antibodies for 30 minutes at 4 °C. The antibodies used were mouse CD3 (1:100, clone: 500A2, V500, San Jose, CA, USA), CD4 (1:250, clone: GK1.5, FITC, eBioscience, CA, USA), CD25 (1:250, clone: PC61.5, PerCP-Cyanine5.5, eBioscience), ROR-γt (1: 100, clone: B2D, APC, eBioscience), Foxp3 (1:100, clone: 150D, PE, eBioscience), F4/80 (1:100, clone: BM8, PE-Cyanine7, eBioscience), Cd11b (1:100, clone: eFluor^®^ 450, M1/70, eBioscience), TLR4 (1:50, clone: UT41, AF488, eBioscience), CD206 (1:100, clone: C068C2, FITC, Biolegend, San Diego, USA). Spectral overlap was corrected using single-stained compensation controls. Doublets were excluded using FSC-H/FSC-W and SSC-H/SSC-W gating during analysis to enable single-cell evaluation, and consistent gating strategies were applied uniformly across all experiments. Data were collected using a FACSVerse flow cytometer (BD Biosciences) and analyzed using FlowJo software (Tree Star, San Carlos, CA, USA).

### Cell culture and treatment

The cell lines in this study were selected to investigate specific mechanistic aspects of intestinal pathophysiology. Caco-2 cells were used to model the intestinal epithelial barrier, while HCT-116-Dual™ and HT29-Lucia™ AhR cells were employed to evaluate transcriptional regulation of NF-κB, IRF, and AhR. RAW264.7 macrophages and HL-60 cells were utilized to study myeloid polarization and innate immune responses, respectively. Caco-2, RAW264.7 (ATCC, Manassas, VA, USA), HCT-116-Dual™, and HT29-Lucia™ AhR cells (InvivoGen, San Diego, CA, USA) were maintained in Dulbecco’s Modified Eagle’s Medium (DMEM; HyClone, Logan, UT, USA) supplemented with 10% fetal bovine serum (FBS) and 1% penicillin-streptomycin. HL-60 cells (ATCC) were cultured in RPMI 1640 medium containing 20% FBS and 1% penicillin-streptomycin. HL-60 differentiation into neutrophil-like cells was confirmed by morphological changes (reduced size and nuclear condensation) after 7 days of 1.25% DMSO treatment. All cells were cultured in a humidified incubator at 37 °C with 5% CO_2_.

Changes in transcriptional activation of NF-κB and IRF were evaluated in HCT-116 cells, while that of aryl hydrocarbon receptor (AhR) was evaluated in HT-29 reporter cells. For the reporter assays, HCT116-Dual™ or HT29-Lucia™ AhR cells were seeded at a density of 5 × 10^4^ cells per well in 96-well plates. After a 30-minute pre-incubation with the test drugs, inflammatory activation was induced using specific stimuli as follows: 100 ng/ml IL-1β for 18 h (for NF-κB), 100 ng/ml poly(dA:dT) for 24 h (for IRF), and 5 μg/ml FICZ for 18 h (for AhR). RAW264.7 macrophages were seeded at a density of 5 × 10^4^ cells per well in 12-well plates and then treated with lipopolysaccharide (LPS), tumor necrosis factor-alpha (TNF-α), or phorbol 12-myristate 13-acetate (PMA) at 100 ng/ml for 2 h to induce activation. Changes in the expression of genes encoding chemokines and M2 macrophage markers were analyzed. The drugs were prepared as 1 000 × stock solutions dissolved in dimethyl sulfoxide (DMSO). Tofacitinib was used at a concentration of 0.32 µg/ml, while 5-ASA (229.7 µg/ml) served as the comparative control drug.

Details on FITC-dextran permeability, transepithelial electrical resistance (TEER) assay, flow cytometric analysis, and quantitative reverse-transcription polymerase chain reaction (qRT-PCR) are described in the Supporting Information.

### FITC-dextran permeability and transepithelial electrical resistance assays

Caco-2 cells were seeded at a density of 2.5 × 10^5^ cells/well into the upper chamber of a 12-well Transwell plate (Corning, NY, USA), with the lower chamber filled with culture medium. After 20 days of culturing to allow for the formation of tight junctions, the cells were washed and then exposed to medium containing the drug in the upper chamber. Following 30 min of incubation, an inflammatory response was induced by treatment with 100 ng/ml tumor necrosis factor-alpha (TNF-α) for 24 hours. To quantify the effects of the drug on tight junctions, FITC-dextran permeability and transepithelial electrical resistance (TEER) assays were performed. For the TEER measurements, a Millicell-ERS2 Volt-Ohm Meter (Merck, MA, USA) was used to assess the electrical resistance between the upper and lower chambers. The readings were adjusted by subtracting values from a well without a sample, then multiplied by the surface area of the Transwell membrane and expressed as ohms per square centimeter (Ω/cm²). For FITC-dextran permeability measurements, the upper chamber of the Transwell plate was filled with 200 µL of FITC-dextran (1 mg/ml) and cultured for 2 hours. The lower chamber medium was collected, and then the fluorescence intensity was measured using a microplate reader (Fluorometer, Varioskan) with excitation and emission wavelengths of 490 and 508 nm, respectively. For the FITC-dextran permeability assessment, the fluorescence values of the negative control group were used as the baseline, and fold changes were calculated accordingly. For the TEER calculations, a provided formula was used, with the negative control values normalized to one for the fold change representation.

### Quantitative reverse-transcription polymerase chain reaction

Total RNA (2 μg), extracted from either the proximal colons or cells using Ribospin™ II (GeneAll, Daejeon, South Korea), was used for complementary DNA (cDNA) synthesis using the High-Capacity cDNA Reverse-Transcription Kit (Thermo Fisher Scientific). The synthesized cDNA was then used for quantitative real-time polymerase chain reaction (qRT-PCR) with the Power SYBR™ Green PCR Master Mix (Thermo Fisher Scientific) on either the StepOnePlus™ Real-Time PCR System (Thermo Fisher Scientific) or the QuantStudio 3 Real-Time PCR Instrument (Thermo Fisher Scientific). The amplification protocol included an initial denaturation step at 95 °C for 30 seconds, followed by 45 cycles of 30 seconds at 59 °C–61 °C and 40 seconds at 72 °C. Gene expression levels were normalized to the housekeeping gene for β-actin to determine relative expression. The results were analyzed using the *ΔΔ*CT method. The specific primer sequences used are listed in **Supplementary Tables S1** and **S2**.

### Immunofluorescence staining

Cells or deparaffinized 4-μm tissue sections were fixed with 10% formalin (pH 7.4), permeabilized with 0.5% Triton X-100 in PBS (PBS-T), and blocked with serum-free protein block (Dako #X0909, Carpinteria, CA, USA). The slides were then incubated overnight at 4 °C with the following primary antibodies: MUC2 (1:2,000, Abcam, Cambridge, UK), Ly6G (1:200, BD Biosciences, San Jose, CA, USA), p-ERM (1:200, Cell Signaling Technology, Inc., Danvers, MA, USA), and ZO-1 (1:200, EnoGene Biotech, NY, USA) or with fluorescent label-conjugated antibodies. Then, the slides were washed thrice with PBS-T and incubated with Alexa Fluor-488, Alexa Fluor-555, or Alexa Fluor-633-conjugated secondary antibodies (Thermo Fisher Scientific, San Jose, CA, USA) for 30 min at room temperature. Cell nuclei were counterstained with DAPI (blue) (Thermo Fisher Scientific). Images were acquired using either light microscopy (Olympus BX41; Olympus Optical, Tokyo, Japan) or confocal microscopy (Carl Zeiss LSM 700, Prenzlauer, Berlin, Germany). All images were acquired using identical microscope settings (including exposure time, gain, and illumination intensity) across all experimental groups. Image quantification was performed using the same analysis parameters and thresholds for all samples.

### Statistical analysis

The data are presented as means ± standard error of the means (SEM) for independent biological replicates (n ≥ 3) or representative data. Statistical analysis was performed using Prism 9.0 software (GraphPad Inc., San Diego, CA, USA). Student’s t-test, one-way or two-way ANOVA, or Kruskal-Wallis test were used as appropriate, following normality and homogeneity of variance testing. A *p*-value < 0.05 was considered statistically significant.

## Results

### CU104 prevents DNBS-induced colitis in mice

**Figure 1:**
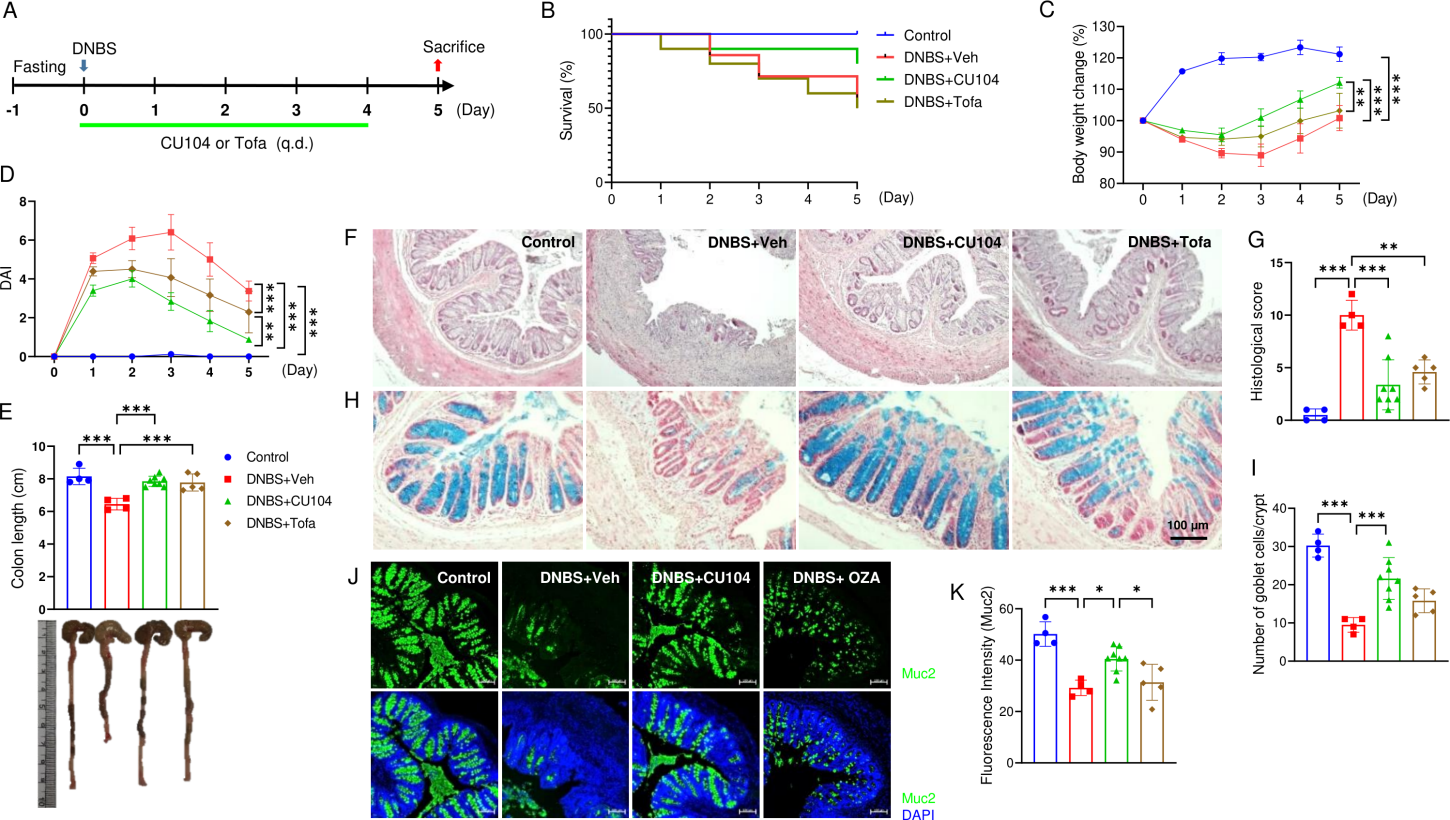
CU104 exhibits superior suppression of disease activity compared with tofacitinib in the DNBS-induced colitis model. **(A)** Experimental scheme. CU104 (10 mg/kg) and tofacitinib (30 mg/kg) were administered orally once daily. **(B)** During the experiment, three, two, and five mice died in the vehicle-treated, CU104-treated, and tofacitinib-treated groups, respectively. Kaplan-Meier plots were used to analyze survival, with overall survival compared using a log-rank test. **(C)** Body weight change. The initial body weight at the start of the experiment, at Day 0, was set as 100%. **(D)** Disease activity index (DAI). **(E)** Representative image of colon length. **(F)** Representative H&E staining images of the colon (Scale bar, 100 μm). **(G)** Histological score of the colon. **(H)** Representative Alcian Blue staining image of the colon. **(I)** Goblet cell number in the crypt. **(J)** Representative mucin staining images of the colon. **(K)** Densitometric analysis of mucin staining. Each group consisted of 4−8 mice. The data are presented as means ± SEM. Significance was indicated by *p < 0.05, **p < 0.01, and ***p < 0.001 using ANOVA with Tukey’s multiple comparisons test. No significance (n.s) between groups was not indicated. Vehicle, vehicle treatment; CU104, CU104 treatment; Tofa, tofacitinib treatment.

To assess their efficacy against DNBS-induced colitis, CU104 (10 mg/kg) and tofacitinib (30 mg/kg), a JAK inhibitor used as the control drug (21, 22), were orally administered once daily (**Figure 1A**). We quantified survival, body weight change, disease activity index (DAI), colon length, histopathology, goblet cell abundance, and mucin production. CU104 increased survival. While the vehicle and tofacitinib groups had survival rates of 57% and 50%, respectively, the CU104-treated group exhibited an 80% survival rate (**Figure 1B**). The vehicle-treated group exhibited the most substantial body weight loss (**Figure 1C**; **Supplementary Table S3**) and the most severe disease activity (**Figure 1D**; **Supplementary Table S4**), including changes in stool consistency and fecal blood on day 3, while these indices were comparatively reduced in the drug-treated groups. Notably, CU104 provided significant protection against overall body weight loss and disease activity compared to the vehicle- or tofacitinib-treated groups. In the vehicle-treated group, there was noticeable colon shortening and edema compared with the control group, whereas the CU104 and tofacitinib groups exhibited a statistically significant reduction in colon shortening and edema (*p* < 0.001: **Figures 1E, F**). To investigate inflammation and structural changes, we assessed the histopathological scores using H&E-stained colon tissue sections. The vehicle-treated group exhibited significant epithelial loss, crypt disruption, and inflammatory cell infiltration in the colon (**Figures 1F, G**), while the histological damage and inflammation were considerably attenuated in the CU104 and tofacitinib groups. Alcian Blue staining, which specifically targets acidic mucopolysaccharides such as those secreted by goblet cells (23), revealed a significant reduction in goblet cell numbers in the vehicle-treated group (**Figures 1H, I**), which was mitigated in the CU104-treated group. Furthermore, the average value of goblet cell depletion in the CU104-treated group was lower than that in the tofacitinib-treated group, although the difference was not statistically significant. Additionally, Mucin 2 immunostaining showed higher mucin levels in the CU104-treated group than in the vehicle-treated DNBS group (23.7 ± 2.4 vs 11.4 ± 0.8, mean ± SEM; **Figures 1J, K**).

**Figure 2:**
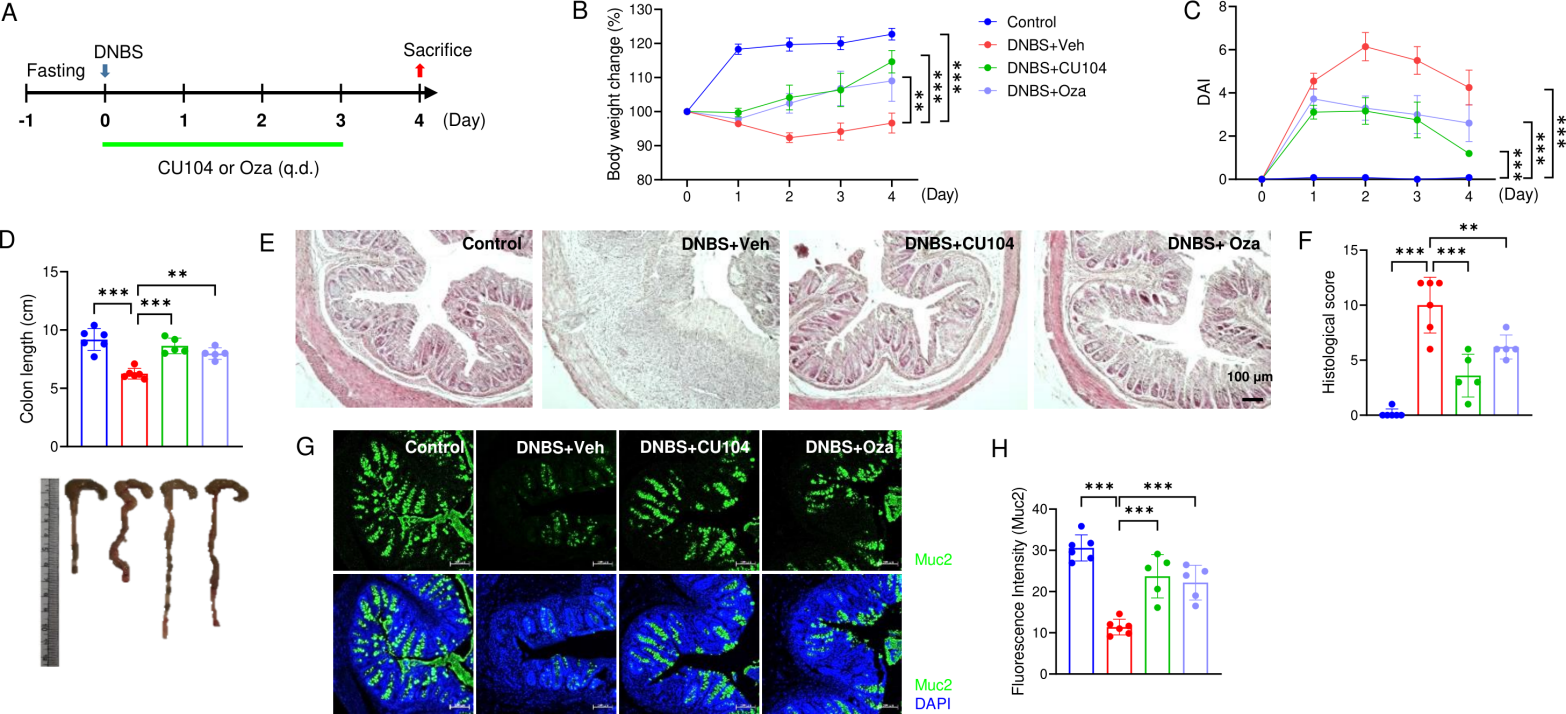
CU104 ameliorates DNBS-induced colitis comparable to ozanimod. **(A)** Experimental scheme. CU104 (10 mg/kg) and ozanimod (1 mg/kg) were administered orally once daily. **(B)** Body weight change. **(C)** Disease activity index (DAI). **(D)** Representative image of colon length. **(E)** Representative H&E staining image of the colon. **(F)** Histological score of the colon. **(G)** Representative mucin staining images of the colon. **(H)** Densitometric analysis of mucin staining. Each group consisted of five mice. The data are presented as means ± SEM. Significance was indicated by **p < 0.01, and ***p < 0.001 using ANOVA with Tukey’s multiple comparisons test. CU104, CU104 treatment; Oza, ozanimod treatment.

The protective effect of CU104 against DNBS-induced colitis was further validated in an independent experiment and compared with ozanimod, a lymphocyte migration blocker used to treat UC (18) (see Methods for dosing and administration). To reduce mouse mortality, the mice were euthanized a day earlier than in the previous experiment (**Figure 2A**). Body weight loss, as well as DAI, was notably decreased in the CU104- and ozanimod-treated groups compared with the vehicle-treated group (**Figures 2B, C**; **Supplementary Tables S5**, **S6**). At the experiment’s endpoint, the shortening of colon length and edema were remarkably reduced in the CU104- and ozanimod-treated groups compared to the vehicle-treated group (**Figures 2D, E**). Histological analysis revealed that the CU104-treated group exhibited a considerable decrease in histopathology compared with the vehicle-treated group (3.6 ± 0.9 vs 10.0 ± 1.0, mean ± SEM). This decrease was comparatively lower than that in the ozanimod-treated group (6.2 ± 0.5, mean ± SEM), although the difference was not statistically significant (**Figures 2E, F**). Similarly, CU104 enhanced the mucin-secreting capacity of goblet cells in the context of DNBS-induced inflammation, surpassing the effects observed with ozanimod (**Figures 2G, H**).

Based on these findings, the therapeutic efficacy of CU104 in the DNBS-induced colitis model was comparable to that of the JAK inhibitor tofacitinib or the S1P receptor agonist ozanimod.

### CU104 regulates the expression of genes related to barrier function, immune cell trafficking, and tissue remodeling in DNBS-induced colitis

**Figure 3:**
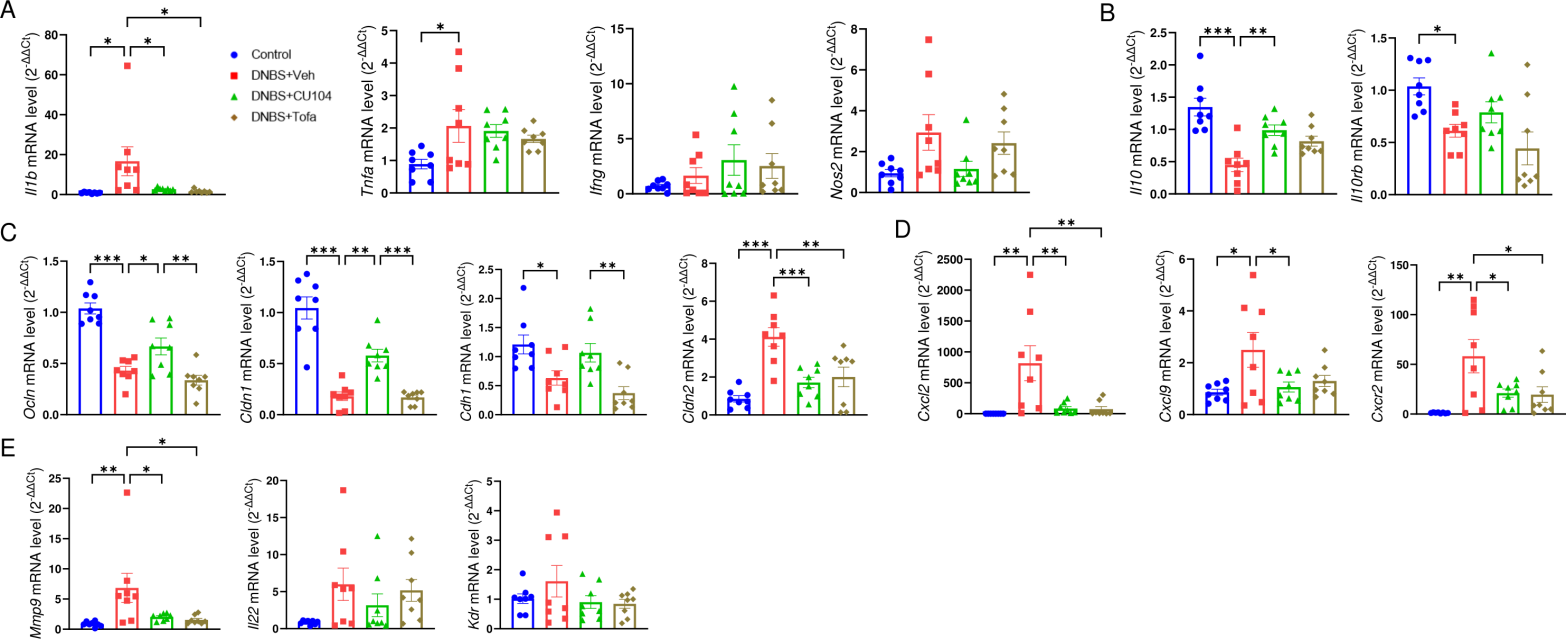
CU104 efficiently protects the leaky epithelial barrier, suppresses inflammation, and induces IL-10 in colitis. The expression of genes encoding immune modulatory molecules, tight junction proteins, chemokines, and factors involved in immune cell rolling and tissue remodeling in the colon was profiled using quantitative reverse-transcription PCR (qRT-PCR). Gene expression profiles of **(A)** proinflammatory molecules (*Il1b*, *Tnfa*, *Ifng*, and *Nos2*), **(B)** anti-inflammatory molecules (*Il10* and *Il10rb*), **(C)** junction proteins (*Ocln*, *Cldn1*, *Cldn2*, and *Cdh1*), **(D)** chemokines (*Cxcl2*, *Cxcl9*, and *Cxcr2*), and **(E)** tissue remodeling factors (*Mmp9*, *Il22*, and *Kdr*). Each group consisted of eight mice. The data are presented as mean ± SEM. Significance was indicated by *p < 0.05, **p < 0.01, and ***p < 0.001 using ANOVA with Dunnett’s T3 multiple comparison test. No significance (n.s) between groups was not indicated. CU104, CU104 treatment; Tofa, tofacitinib treatment.

To evaluate the expression of genes related to cytokines, barrier function, immune cell trafficking, and tissue remodeling, including vascularization, we analyzed the changes in their transcript levels in the colon using qRT-PCR. As previously reported (24), the gene expressions of proinflammatory cytokines such as IL-1β (*Il1b*), TNF-α (*Tnfa*), and IFN-γ (*Ifng*), and inducible nitric oxide synthase (*Nos2*) were elevated, while that of the anti-inflammatory cytokine (*Il10* and *Il10rb*) was reduced in the DNBS+Veh group compared with the control group (**Figures 3A, B**). We also observed that the expression of genes encoding tight junction and adherent proteins, including occludin (*Ocln*), claudin 1 (*Cldn1*), and E-cadherin (*Cdh1*), was reduced, but that of claudin 2 (*Cldn2*), a pore-forming tight junction protein, was increased in the DNBS + Veh group. The expression of *Il1b* was substantially downregulated in the CU104- and tofacitinib-treated groups, but no substantial changes were seen in other cytokines or *Nos2*. Notably, the expression of *Ocln*, *Cldn1*, and *Cdh1*, along with *Il10* was increased, while *Cldn2* expression was suppressed in the CU104-treated group, but not in the tofacitinib-treated group (**Figure 3C**). These results suggest that the anticolitic effect of CU104 can, at least partially, result from improving intestinal barrier function independently of the suppression of proinflammatory cytokines.

DNBS treatment enhanced the expressions of genes related to neutrophil chemotaxis and rolling (*Cxcl2*, *Cxcl9*, and *Cxcr2*), as well as tissue remodeling, including matrix metalloproteinase-9 (*Mmp9*), IL-22 (*Il22*), and vascular endothelial growth factor receptor 2 (*Kdr*). CU104 treatment was associated with reduced expression of these injury-/inflammation-associated transcripts (**Figures 3D, E**).

These results support the notion that CU104 improves colitis by affecting innate immune responses and immune cell recruitment, rather than directly affecting immune modulation.

### CU104 alleviates colitis in IL-10 KO mice

**Figure 4:**
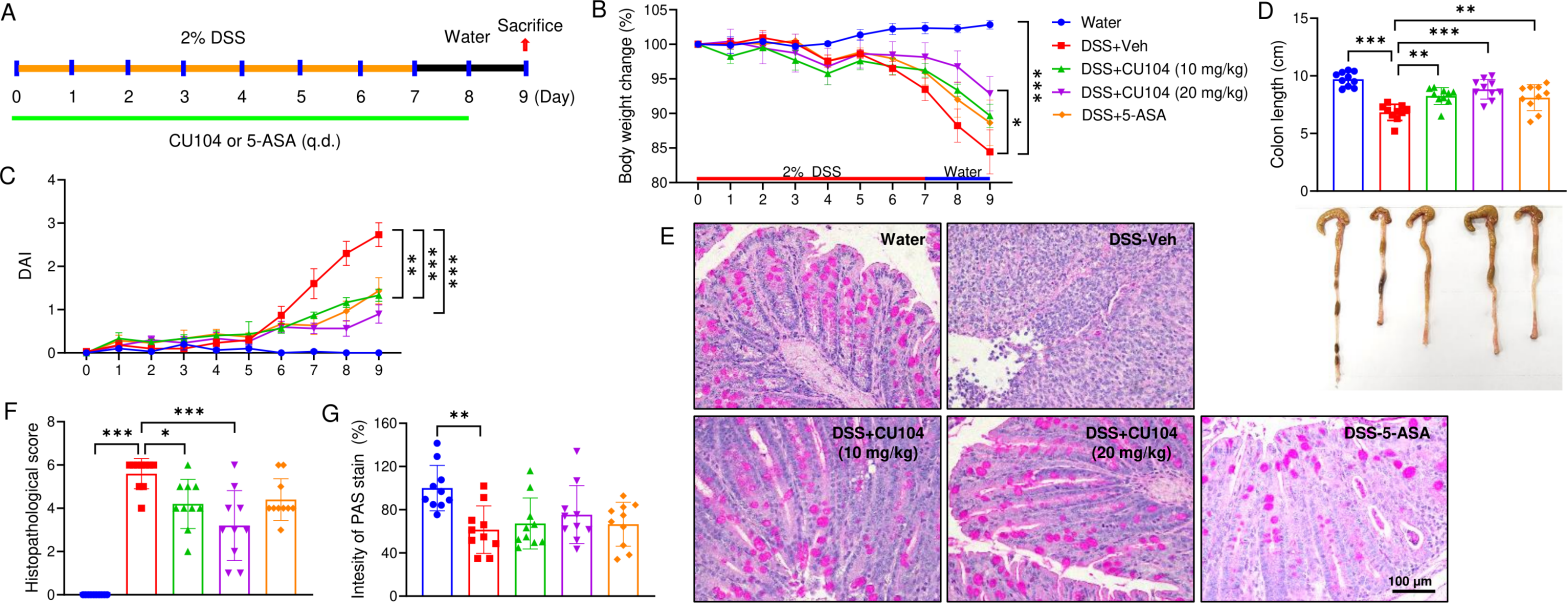
CU104 ameliorates DSS-induced colitis in IL-10 KO mice. **(A)** Experimental design. Colitis was induced in IL-10 knockout (*Il10*^-/-^) C57BL/6 mice by treating them with 2% DSS for seven days and then with normal drinking water for two days. **(B)** Body weight change. **(C)** Disease activity index (DAI). **(D)** Representative image showing colon length. **(E)** Representative sections of PAS stain. **(F)** Histological score. **(G)** Densitometric analysis of PAS stain. Each group consisted of ten mice. The data are presented as means ± SEM. For multiple comparisons, statistical significance was assessed using one-way ANOVA followed by Tukey’s *post-hoc* test. *p < 0.05, **p < 0.01, and ***p < 0.001. DSS, dextran sodium sulfate treatment; CU104, CU104 treatment; 5-ASA, 5-ASA treatment.

Given the observed CU104-mediated increase in IL-10 levels in the DNBS-induced colitis model, we next assessed whether CU104 retains anticolitic efficacy under IL-10–deficient conditions by testing CU104 in DSS-challenged IL-10 knockout mice, using 5-ASA (25) as a reference treatment (**Figure 4A**). When colitis developed, the mice exhibited diarrhea, resulting in a decrease in body weight (**Figure 4B**; **Supplementary Table S7**). The body weight was notably decreased in the DSS+Veh group compared to the Water group (84.4 ± 3.2% vs 102.8 ± 0.6% on Day 9), but this decrease was substantially mitigated in the DSS+CU104 (20 mg/kg) group (92.9 ± 2.5% on Day 9). The occurrence of fecal blood and diarrhea, as well as colon length shortening, was decreased in the CU104- and 5-ASA groups (**Figures 4C, D**; **Supplementary Table S8**). Histopathological analysis showed that the colons of the DSS+Veh group exhibited loss of epithelial lining, few or absent crypts, and extensive infiltration of immune cells, which were not observed in the Water group (**Figure 4E**). The inflammatory cell infiltration was markedly changed while epithelial cell integrity was improved following CU104 treatment. Simultaneously, the DSS+CU104 (10 mg/kg) and DSS+CU104 (20 mg/kg) groups showed a considerably decreased histopathological score compared with the DSS+Veh group on Day 9 (1.3 ± 0.1 and 0.9 ± 0.2 vs 2.7 ± 0.3, respectively; **Figures 4E, F**). Mucin production from goblet cells was most likely increased in the CU104-treated groups (67.3 ± 7.5 for CU104 (10 mg/kg) and 75.5 ± 8.5 for CU104 (20 mg/kg) compared with the DSS+Veh group (61.5 ± 6.9)), without any significant enhancement in the other drug-treated groups (66.6 ± 6.4, mean ± SEM; **Figure 4G**), implying that IL-10 deficiency may affect mucin production, as reported previously (26, 27).

**Figure 5:**
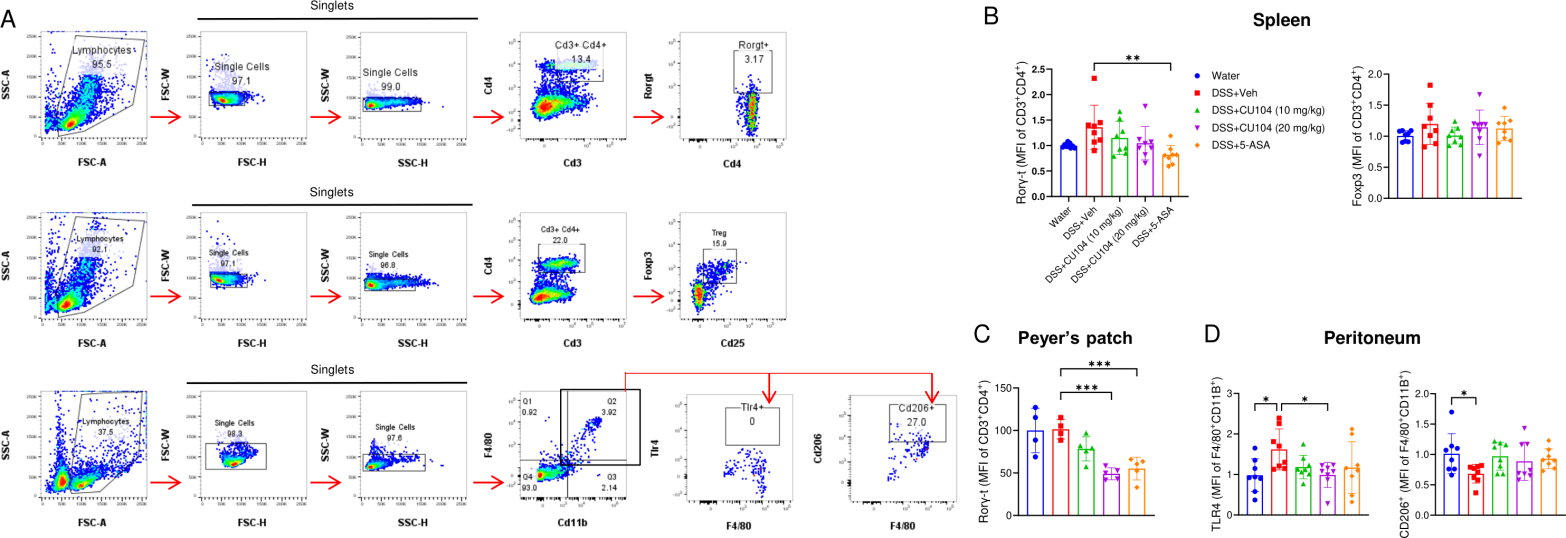
CU104 affects immune cell recruitment rather than directly influencing the modulation of adaptive immune cells. Colitis was induced in IL-10 knockout (*Il10*^-/-^) C57BL/6 mice by treating them with 2% DSS for seven days and then with normal drinking water for two days. **(A)** Representative flow cytometry gating strategy. **(B-D)** Cells were extracted from the spleen, Peyer’s patches, and peritoneum. Flow cytometric analysis was performed for Th17 cells (Rorγ-t), Treg cells (Foxp3), M1 macrophages (TLR4), and M2 macrophages (CD206). Median fluorescence intensity (MFI) is indicated. Each group consisted of 4−8 mice. The data are presented as means ± SEM. Statistical significance was assessed using one-way ANOVA followed by Dunnett’s multiple comparisons test. *p < 0.05, **p < 0.01, and ***p < 0.001. DSS, dextran sodium sulfate treatment; CU104, CU104 treatment; 5-ASA, 5-ASA treatment.

We analyzed the changes in Th17 and Treg cells from the spleens and Peyer’s patches of IL-10 KO mice, as well as macrophages from the peritoneum, to understand the alterations in innate and adaptive immune responses during colitis (**Figure 5A**). Flow cytometric analyses revealed an increasing trend of the Th17 cell marker (Rorγt) and the Treg cell marker (Foxp3) in splenic helper T cells (CD3^+^CD4^+^ cells) from DSS-treated mice compared with the water group (**Figure 5B**). However, we observed no statistically significant increase in Rorγt in helper T cells of Peyer’s patches in the DSS-treated group compared with the Water group (**Figure 5C**). In contrast, we observed a marked increase in the M1 macrophage marker TLR4 in the DSS+Veh group compared with the Water group (1.62 ± 0.18 vs 0.98 ± 0.14), while the M2 macrophage marker CD206 was decreased (0.68 ± 0.05 vs 1.01 ± 0.12; **Figure 5D**). The Th17 cells were notably downregulated in the spleen and Peyer’s patches of the 5-ASA-treated group, whereas the same results were observed only within the Peyer’s patches in the CU104-treated group. In contrast to T-cell analyses, the levels of the M1 macrophage marker (0.99 ± 0.11) were considerably reduced in the peritoneal cavity cells from the CU104-treated group (20 mg/kg), along with an increased trend in the M2 macrophage markers (0.89 ± 0.11), compared with the DSS+Veh group (**Figures 5B−D**).

These data indicate that CU104 mitigates colitis in IL-10 KO mice, independent of IL-10 or anti-inflammatory immune cells, such as Treg cells, by affecting innate immune cells, including macrophages.

### CU104 directly affects intestinal barrier integrity and immune cell trafficking in an IL-10-independent manner

**Figure 6:**
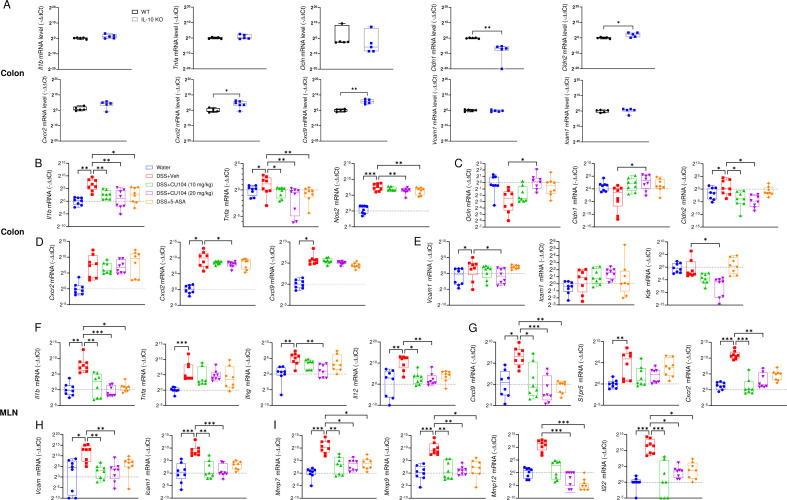
CU104 affects immune cell recruitment and tissue remodeling independently of IL-10. **(A)** IL-10 KO mice exhibits increased intestinal barrier permeability and chemokines. The differences in gene expression profiles in the colon between DSS-untreated wild-type mice and IL-10 knockout (KO) mice were assessed using quantitative real-time PCR (qRT-PCR). The gene expression profiles of cytokines (*Il1b* and *Tnfa*), tight junction proteins (*Ocln*, *Cldn1*, and *Cldn2*), chemokine receptor/chemokine (*Cxcr2*, *Cxcl2*, and *Cxcl9*), and immune cell rolling factors (*Vcam1* and *Icam1*). **(B-I)** Gene expression related to proinflammatory cytokines and NO production (*Il1b*, *Tnfa*, *Nos2*, *Ifng*, and *Il12*), tight junction proteins (*Ocln*, *Cldn1*, and *Cldn2*), immune cell recruitment (*Cxcr2*, *Cxcl2*, *Cxcl9*, *Vcam1*, *Icam1*, and *S1pr5*), angiogenesis (*Kdr*), and tissue remodeling (*Mmp7*, *Mmp9*, *Mmp12*, and *Il22*) in the colons **(B-E)** and mesenteric lymph nodes **(F-I)** were profiled using qRT-PCR. Each group consisted of 4−8 mice. The data are presented as means ± SEM. Statistical significance was assessed using one-way ANOVA followed by Dunnett’s multiple comparisons test. *p < 0.05, **p < 0.01, and ***p < 0.001. DSS, dextran sodium sulfate treatment; CU104, CU104 treatment; 5-ASA, 5-ASA treatment.

We analyzed the changes (by qRT-PCR) in gene expressions related to proinflammatory cytokines, barrier function, immune cell recruitment, and tissue remodelling in the colons of wild-type and IL-10 knockout (KO) mice. Interestingly, we observed marked changes in the expressions of genes related to tight junctions (notably reduced *Cldn1* but elevated *Cldn2*), chemokines, and chemokine receptors (substantially elevated *Cxcl2* and *Cxcl9*) in the colons of DSS-untreated, naïve IL-10 KO mice compared with wild-type mice (**Figure 6**). These results are consistent with previous reports on Treg dysfunction in IL-10 KO mice (28), which contributed to the characteristically elevated inflammatory response in these mice under normal conditions. Upon DSS treatment, consistent with previous reports (24), elevated gene expression for proinflammatory cytokines (such as *Il1b* and *Tnfa*), *Nos2*, chemokines (such as *Cxcr2*/*Cxcl2* and *Cxcl9*), and vascularization molecules (such as *Vcam1*, *Icam1*, and *Kdr*) was detected in the colon (**Figures 6B–E**). We noticed that DSS treatment drastically reduced gene expressions of genes associated with tight junction proteins (*Ocln* and *Cldn1*), while notably increasing *Cldn2* expression (**Figure 6F**). Both the CU104- and 5-ASA-treated groups exhibited significant downregulation in the expressions of *Il1b, Tnfa*, and *Nos2*. Notably, the expressions of *Ocln* and *Cldn1* were considerably increased, and that of *Cldn2* was decreased only in the CU104-treated group, and not in the 5-ASA group. This observation suggests that CU104 can enhance intestinal barrier function independently of IL-10. Remarkably, only the DSS+CU104 (20 mg/kg) group exhibited a statistically significant difference in the expression of *Vcam1*, which is specifically expressed in endothelial cells within blood or lymphatic vessels, and *Cxcl2* compared with the DSS+Veh group. This difference was not observed in the DSS + 5-ASA groups. Furthermore, Kdr expression decreased from DSS+Veh (11.47 ± 10.47) to the DSS+CU104-treated groups (0.08 ± 0.03 and 0.02 ± 0.01, mean ± SEM), corresponding to a 99–100% reduction (**Figures 6D, E**). These findings underscore that CU104 treatment was associated with significant downregulation of genes related to tissue remodeling, including immune cell recruitment and angiogenesis, accompanied by loss of vascular integrity (29), thereby contributing to reduced immune cell recruitment and protecting barrier function. Enlarged mesenteric lymph nodes (MLNs) were more commonly observed in IL-10 KO mice with spontaneous colitis than in wild-type mice (data not shown). To investigate the regulatory effects of CU104 on genes related to immune cell recruitment and infiltration, we assessed the gene expression profiles in MLNs. We observed increased proinflammatory cytokine gene expressions in MLNs of DSS-treated mice compared to the Water group (**Figure 6F**). Similar to the gene expressions in the colon, the expressions of *Il1b*, *Ifng*, and *Il12* (signatures of Th1 cells) were substantially reduced in the CU104-treated group. *Il1b* expression was reduced from DSS+Veh (1080.07 ± 874.75) to DSS+CU10 (15.01 ± 7.11) and DSS+CU20 (1.22 ± 0.49). Conversely, no marked changes in *Ifng* and *Il12* expression were observed in the 5-ASA-treated group compared with the DSS+Veh group, suggesting that the primary therapeutic target of CU104 may differ from that of 5-ASA. CU104 exhibited marked suppression of gene expressions related to immune cell chemotaxis and rolling (*Cxcl9*, *S1pr5*, and *Cxcr2*), adhesion (*Vcam1* and *Icam1*), and infiltration (*Mmp7*, *Mmp9*, and *Mmp12*), as well as IEC wound repair (*Il22*) (**Figures 6F–I**).

Taken together, these results suggest that CU104 improves colitis by affecting the recruitment and infiltration of immune cells, as well as barrier function, rather than directly affecting immune modulation, independently of cytokines.

### CU104 regulates the IRF and NF-κB signaling pathways but not the AhR pathway

**Figure 7:**
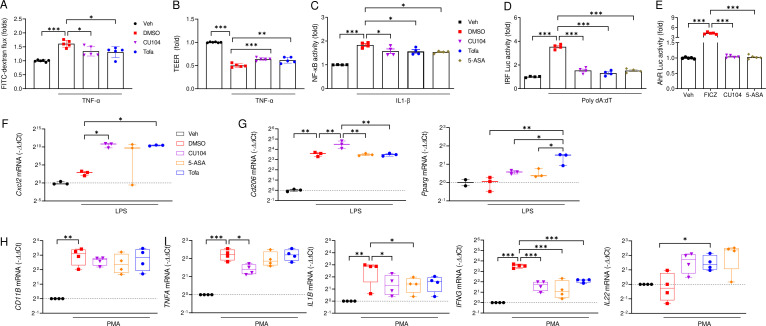
CU104 ameliorates intestinal barrier dysfunction by suppressing the NF-κB/IRF pathway and modulates innate immune cells. Caco-2 cells were pretreated with vehicle, CU104 (10 μg/ml), 5-ASA (15 μM), or tofacitinib (1 μM) (30 min) followed by TNF-α (100 ng/ml, 24 h), and FITC-dextran permeability was assessed. **(A)** FITC-dextran permeability and **(B)** Transepithelial electrical resistance (TEER) assays. **(C, D)** NF-κB and IRF reporter HCT-16 cells were stimulated with IL-1β or poly (dA:dT) for 24 h or 12 h, respectively, to induce the transcriptional activation. **(E)** 6-Formylindolo [3,2-b] carbazole (FICZ) was used to activate AhR transcription in HT29-Lucia™ AhR Cells. NF-κB activity was assessed by measuring absorbance at OD_633_, while IRF and AhR activities were quantified using luciferase activity. **(F−I)** Gene expression of a chemokine (*Cxcl2* and *Cxcl8*), M2 markers (*Cd206* and *Pparg*), a neutrophil marker (*CD11B*), or cytokines (*TNFA*, *IL1B*, *IFNG*, and *IL22*) in lipopolysaccharide (LPS)-stimulated RAW264.7 cells **(F, G)** and phorbol 12-myristate 13-acetate (PMA)-stimulated neutrophil-like HL-60 cells **(H, I)**. The data are presented as means ± SEMs of at least three independent experiments. Statistical significance was assessed using one-way ANOVA followed by the Tukey multiple comparisons *post-hoc* test. *p < 0.05, **p < 0.01, and ***p < 0.001. Veh, phosphate-buffered saline treatment; DMSO, dimethyl sulfoxide treatment treatment; CU-104, CU104 treatment; 5-ASA, 5-ASA treatment; Tofa, tofacitinib treatment.

Upregulation of the IL-1β and IFN-γ signal pathways, resulting from elevated Th1 and Th17 cells and IL-10 deficiency (30), can increase the permeability of IECs. To investigate whether CU104 enhances the IEC barrier function *in vitro*, we first conducted a FITC-dextran permeability assay and TEER measurement in Caco-2 cells using a Transwell system. TNF-α treatment increased FITC-dextran permeability to 1.61 ± 0.06 (vs 1.00 ± 0.01 in Veh), whereas CU104 reduced it to 1.35 ± 0.08 (**Figure 7A**). TEER decreased from Veh (1.00) to 0.50 ± 0.02 with TNF-α (DMSO) and recovered to 0.64 ± 0.01 with CU104 (**Figure 7B**). In the TNF-α-treated group, FITC-dextran permeability increased substantially, accompanied by a significant decrease in TEER value, indicating the disruption of tight junctions (**Figures 7A, B**). CU104 treatment prevented increased FITC-dextran permeability and restored TEER levels in Caco-2 cells, comparable to those of tofacitinib.

Of the three key transcription factors related to inflammation, NF-κB and IRF are hyperactivated in patients with active IBD, whereas the activity of AhR, which plays a pivotal role in barrier function, is diminished (1, 31). To elucidate the signal pathway protecting the integrity of the intestinal epithelial barrier, including immune responses, we evaluated alterations in these three transcription factors using reporter cells: HCT-116-Dual™ cells for NF-κB and IRF activity, and HT29-Lucia™ AhR cells for AhR activity. We stimulated the HCT-116-Dual™ cells with IL-1β and poly dA:dT to study the activity of NF-κB and IRF, respectively. Furthermore, we investigated AhR induction in HT29-Lucia™ AhR cells after the administration of CU104, tofacitinib, or 5-ASA. In HCT-116 reporter cells, the activity of NF-κB and IRF was increased after treatment with IL-1β and poly dA:dT (**Figures 7C, D**). CU104, tofacitinib, or 5-ASA-treated groups showed a decreased NF-κB and IRF promoter activity compared with the control group (**Figures 7C, D**). However, no observable effect on AhR activity in HT29 reporter cells was found with either CU104 or 5-ASA treatment, although the AhR agonist FICZ did induce AhR activity (**Figure 7E**). These results align with reports showing that CU104 treatment does not notably may contribute to *Il22* expression in the colon of the IL-10 KO mice.

We investigated whether CU104 affects the modulation of innate immune cells, specifically macrophages, which are critical components of innate immunity and IEC barrier function, and act as sentinels against pathogens alongside neutrophils. We stimulated RAW264.7 cells, a mouse macrophage cell line, with LPS to evaluate the changes in gene expression following drug pretreatment. LPS treatment augmented specific chemokine markers (*Cxcl2*) (**Figure 7F**). The expression of these genes was inhibited in the groups treated with CU104, tofacitinib, or 5-ASA. CU104 further increased the expression of *Cd206*, an M2 macrophage marker (**Figure 7G**), consistent with a previous finding in a myocardial ischemia–reperfusion injury model (7). Among the drugs tested, tofacitinib treatment induced the expression of *Pparg* compared with the control.

Next, we explored the impact of CU104 on neutrophils, which are the most abundant immune cells and the pathological indicator of IBD (32). To explore this, we used the HL-60 cell line, which was differentiated into neutrophil-like cells using DMSO. Upon PMA stimulation, the differentiated HL-60 cells exhibited increased gene expression of CD11B (**Figure 7H**), a neutrophil marker (33), and proinflammatory cytokines, such as TNF-α, IL-1β, and IFN-γ (**Figure 7I**). All drugs mitigated the increased expression of *IL1B* and *IFNG* but elevated the expression of *IL22*, a marker associated with IL-22-producing neutrophils. Of the drugs, only CU104 treatment significantly suppressed the expression of *TNFA* and upregulated that of *IL22*.

These findings suggest that CU104 prevents against the aggravation of the intestinal barrier and immune response, including immune cell recruitment, potentially through modulation of IRF and NF-κB signaling. Additionally, CU104 modulates the innate immune response, particularly affecting innate immune cells such as macrophages and neutrophils, and this action appears to be independent of AhR signaling.

### CU104 modulates barrier function and neutrophil trafficking by stabilizing actin dynamics

**Figure 8:**
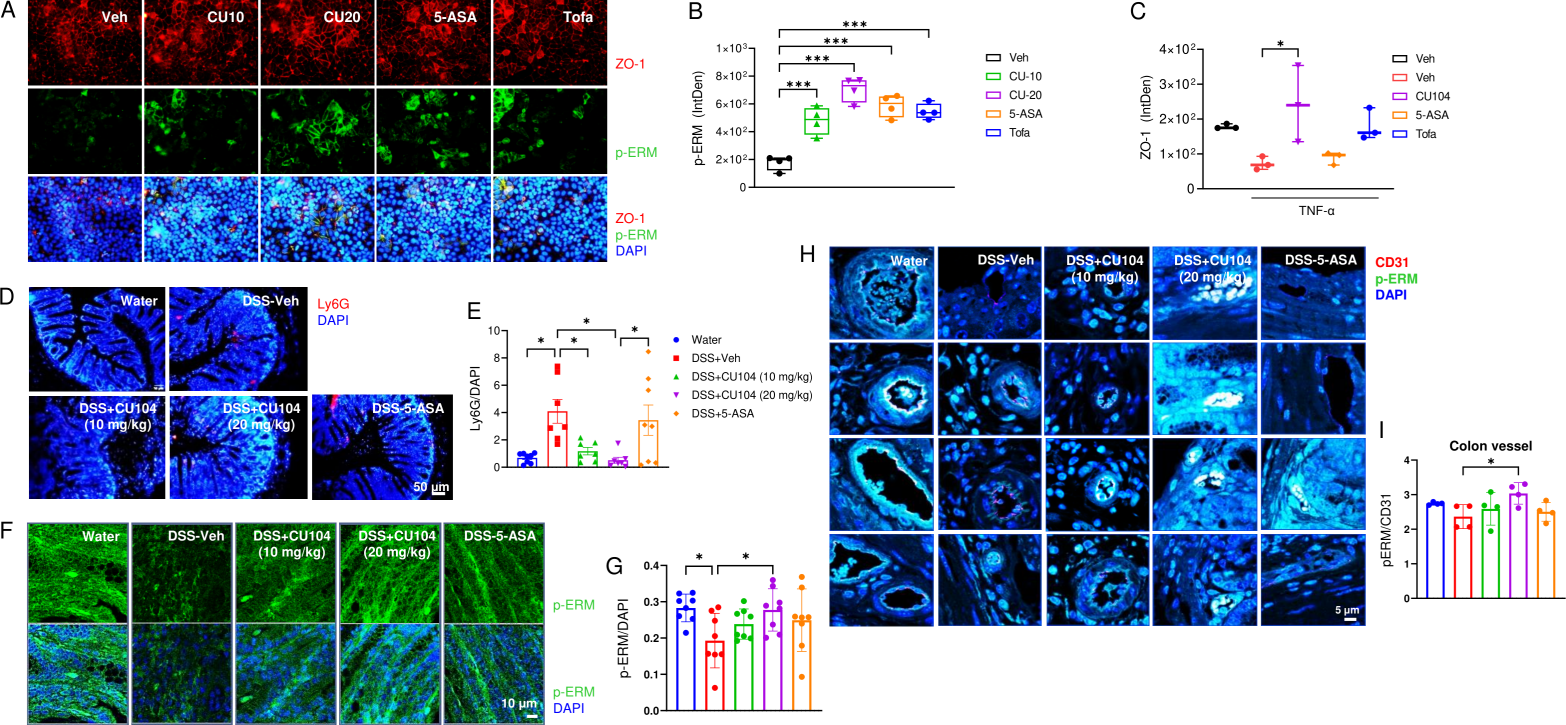
CU104 modulates barrier function and neutrophil trafficking by stabilizing actin dynamics. Caco-2 cells were pretreated with vehicle, CU104 (10 μg/ml), 5-ASA (15 μM), or tofacitinib (1 μM) (30 min) followed by TNF-α (100 ng/ml, 24 h). Cells were immunostained for ZO-1 (red, Alexa 555) and phospho-ezrin/radixin/moesin (p-ERM) (green, Alexa 488). Nuclei were stained with DAPI. **(A)** Representative image of ZO-1 and p-ERM in Caco-2 cells. The data are presented as means ± SEM from at least three independent experiments. **(B)** Densitometric analysis of p-ERM and ZO-1. **(C-H)** Colitis was induced in IL-10 knockout (*Il10*^-/-^) C57BL/6 mice by treating them with 2% DSS for seven days, and then with normal drinking water for two days. **(C, D)** Representative images **(C, E)** and densitometric analysis of Ly6G (neutrophil marker, red) and p-ERM (green) **(D, F)** in the colon, respectively. Ly6G and p-ERM intensities were normalized to that of the DAPI stain (nucleus). **(G, H)** Representative images and densitometric analysis of p-ERM (green, Alexa 488) in the colonic blood vessels. Statistical significance was assessed using one-way ANOVA followed by Tukey’s multiple comparisons *post-hoc* test (n = 8/group). *p < 0.05, and ***p < 0.001. Each group consisted of eight mice. The data are presented as means ± SEM. Veh, phosphate-buffered saline treatment; DMSO, dimethyl sulfoxide treatment; CU-104, CU104 treatment; 5-ASA, 5-ASA treatment; Tofa, tofacitinib treatment; DSS, dextran sodium sulfate treatment.

Rho-associated coiled-coil-containing protein kinase 1 (Rock1) and Ras-associated C3 botulinum toxin substrate 1 (Rac1) are crucial for actin polymerization and stabilization, and for regulating actin dynamics and cell motility (34). In particular, the ROCK1/ERM pathway is crucial for maintaining barrier integrity and regulating neutrophil rolling. As this process involves the phosphorylation of the ERM protein family, we conducted immunofluorescence staining to examine the phosphorylation status of ERM and determine whether CU104 affects cytoskeleton formation and stability through the ERM pathway in Caco2 cells. As expected, TNF-α treatment reduced tight junction protein ZO-1 levels and ERM protein phosphorylation in Caco2 cells (**Figures 8A, B**). CU104 increased ZO-1 intensity to 242.91 ± 63.21 (vs 72.55 ± 11.02 in TNF-α), representing a 235% increase (**Figure 8C**). To verify this phenomenon *in vivo*, we evaluated phospho-ERM levels in the colon of IL-10 KO mice using immunostaining. First, we confirmed that the increased infiltration of neutrophils (Ly6G^+^ cells) into the inflamed region of IL-10 KO mice by DSS was notably reduced in the CU104-treated groups (**Figures 8D, E**). Ly6G^+^ neutrophil signal decreased by 89-97% in DSS+CU104 compared with DSS+Veh (**Figures 8D, E**). Furthermore, we found that phospho-ERM levels were downregulated following DSS administration. Notably, a statistically significant reversal of phospho-ERM levels in colons and vessels was observed in the DSS+CU104 (20 mg/kg) group (0.28 ± 0.02), whereas there was an increased trend in all drug-treated groups (0.24 ± 0.01 for DSS+CU104 (10 mg/kg) and 0.25 ± 0.03 for DSS + 5-ASA) compared with the DSS+Veh group (0.19 ± 0.03; **Figures 8F–I**). These results are similar to the previous findings, which indicated that CU104 activates the Rac signaling pathway in endothelial cells (6).

Collectively, these findings demonstrate that CU104 can modulate barrier integrity, innate immune responses, and the recruitment of immune cells such as neutrophils, which is crucial in the context of IBD, by regulating the ROCK1/ERM pathway, as well as NF-κB and IRF signalling.

## 4 Discussion

We demonstrate that CU104 ameliorates experimental colitis by restoring intestinal barrier function and suppressing inflammation through cytoskeletal stabilization and IRF/NF-κB modulation. Independent of the AhR and IL-10/IL-22 axes, CU104 restricts myeloid cell infiltration by downregulating chemokines and adhesion molecules. These findings suggest that targeting cytoskeleton-mediated immune cell trafficking is a viable therapeutic strategy for IBD. In the DNBS- and DSS-induced colitis models of IL-10 KO mice, CU104 effectively inhibited epithelial leakage and alleviated inflammation. In the DNBS-induced model, CU104 increased mucin production, which is essential for intestinal barrier function, demonstrating its protective effect against colonic damage. However, in the IL-10 KO colitis model, CU104 did not exhibit a clear protective effect on goblet cells, which is consistent with previous findings that IL-10 influences mucin secretion from goblet cells (35). CU104 treatment also improved the expression of proteins regulating the permeability of epithelial and endothelial cells, such as tight junction proteins (Occludin, Claudin-1, and Zo-1), while concurrently downregulating claudin-2, further validating its barrier-enhancing effect (36). To understand the mechanisms underlying the effects of CU104 on barrier function and inflammation, we investigated relevant signalling pathways involving three key transcription factors. IRF and NF-κB are involved in maintaining intestinal barrier function and in the production of proinflammatory cytokines such as TNF-α, IL-1β, and IFN-γ (37). However, AhR plays a barrier-protective role by inducing IL-22 and IL-10 expression (38), and inhibiting proinflammatory cytokine production in T cells (31). The results of the reporter assay in epithelial cells suggested that CU104 restricted cytokine responses and directly regulated tight junction proteins by inhibiting NF-κB and IRF signaling in endothelial cells (8). These findings are consistent with previous studies showing the role of IRF in modulating innate and adaptive immunity (39), and actin cytoskeleton remodeling in endothelial cells (40). However, AhR signaling remained unaffected despite increased IL-22 expression in HL-60 cells. These results suggest that CU104 primarily ameliorates colitis by modulating the IRF pathways, including NF-κB, rather than by influencing AhR signaling. Consistently, CU104 treatment was not associated with *IL22* induction, whereas tofacitinib treatment correlated with significantly higher *IL22* expression in neutrophils. Although IL-22 is generally considered epithelial-protective in acute injury, its expression is also strongly induced by epithelial damage and inflammatory cues. Thus, the reduced IL-22 levels observed with CU104 likely reflect an overall attenuation of injury/inflammation and/or reduced accumulation of IL-22–producing cells in the colon, rather than impaired tissue repair. These findings demonstrate that CU104 protects barrier function independently of IL-10 and AhR signaling and that this effect is associated with changes in actin dynamics.

IL-10, a key anti-inflammatory cytokine, is crucial for regulating immune cells, including macrophages, as well as the Th1/Th17-Treg cell axis. IL-10 also contributes to intestinal barrier function by increasing goblet cell numbers along with proinflammatory cytokines, including IL-1β (41), and its deficiency promoted intestinal barrier permeability (**Figure 6A**). In terms of the pathogenesis, IL-10 KO mice with colitis exhibit upregulated Th1- and Th17-mediated responses, similar to those observed in the DNBS model and in CD (24, 42). Consistently, DSS-treated IL-10 KO mice exhibited markedly elevated expression of the genes encoding IFN-γ and IL-12 in the MLNs, which initiate pathogenic Th1- or Th17-mediated responses. However, under our experimental conditions, CU104 treatment did not inhibit the Th1 and Th17 cell-mediated inflammatory responses in IL-10 KO mice, despite observing notable reductions in most cytokines. This may be due to Treg cell dysfunction observed in naïve IL-10 KO mice (24). As CU104 could significantly suppress Th1/Th17 cell-mediated responses in secondary lymphoid organs (lymph nodes and Peyer’s patches) without modulating adaptive immune cells such as Th1, Th17, and Treg cells, CU104 may exert its effect on innate immune responses or immune cell recruitment via other mechanisms of action. In fact, we observed that the gene expressions of various chemokines were upregulated in naïve IL-10 KO mice (**Figure 6A**). Conversely, CU104 significantly suppressed the expressions of *Cxcl2*/*Cxcr2* and *Cxcl9* in the colons. In particular, CXCL2 is essential for attracting neutrophils through its interaction with CXCR2 receptors on neutrophil surfaces (43), as the CXCL1/CXCL2 released from macrophages controls the initial recruitment of neutrophils into tissues (44). Furthermore, CU104 exerted its suppressive effects only on IL-1β (a potent proinflammatory cytokine primarily produced by innate immune cells (30)) among all the cytokines examined *in vivo*, reflecting its mechanism of inhibiting myeloid cell (neutrophils and macrophages, the predominant immune cells in acute colitis models) recruitment. Further studies using chronic colitis models and human ex vivo models are needed to validate this mechanism.

The observed increase in lymphocyte antigen 6G (Ly6G), a protein primarily expressed in neutrophils, suggests that neutrophils may contribute to the pathogenesis of DSS-induced IL-10 KO colitis and potentially other colitis models. In the DSS-induced IL-10 KO colitis model, the CU104-treated group exhibited reduced neutrophil accumulation and signature gene expression in the inflamed colon and lymph nodes, implying that CU104 can directly affect neutrophils, independent of the anti-inflammatory response by Treg cells in IL-10 KO mice. This direct modulation could contribute to the anticolitic effect by inhibiting immune cell recruitment and polarization, and cytokine production independently of IL-10, despite previous reports showing that CU104 directly affects macrophages and IL-10 (similar to tofacitinib or 5-ASA) by upregulating M2 macrophage-related genes (e.g., *Cd206, Ppar-γ*) in RAW264.7 cells (7).

The secretion of proinflammatory cytokines, chemokines, and MMPs can promote the recruitment, activation, and mobilization of neutrophils, which impair the intestinal mucosa at the target tissue site, thereby triggering disease progression. Chemokines, such as CXCL8 (IL-8), CXCL2, and CXCL9, along with their receptors such as CXCR1, CXCR2, and CXCR3, including sphingosine-1-phosphate receptor 5 (*S1pr5*) (45), mediate neutrophil recruitment. In the early stages of IBD, upregulated VEGF is linked to angiogenesis, potentially promoting increased vascular permeability and immune cell recruitment (46). Furthermore, vascular cell adhesion molecules (VCAM)-1 and ICAM-1, which belong to the immunoglobulin superfamily, mediate the adhesion of leukocytes to vascular endothelium. ICAM-1 is inherently found on the surfaces of leukocytes and endothelial cells in the blood and lymphatic vessels, whereas VCAM-1 is expressed on the membranes of vascular endothelial cells, bone marrow stromal cells, spleen stromal cells, thymic epithelial cells, peripheral LNs, and mesenteric LN high endothelial venules. Consistent with previous findings on CU104 in DSS-induced colitis (10), CU104 markedly dampened the expression of these genes as well as chemokine/chemokine receptors and inflammatory adhesion molecules, underscoring its role in regulating immune cell rolling. ICAM-1-mediated leukocyte adhesion is presumed to govern the initial reorganization of F-actin, endothelial tightening, and barrier maintenance during neutrophil diapedesis (47). Our findings also indicate that CU104 suppressed elevated gene expression of MMPs in MLNs and colons. MMPs, including MMP-9, are produced by various cells, including neutrophils, mononuclear leukocytes, and lymphatic endothelial cells, mediate neutrophil migration (48), and contribute to intestinal barrier damage. Therefore, MMPs act as surrogate markers for calprotectin and disease activity in IBD (49). In this context, the downregulation of MMP genes in the CU104-treated group might be another potential mechanism underlying the improvement in vascular and epithelial leakage as well as neutrophil regulation. Indeed, exploring MMP inhibitors represents a promising avenue for preclinical investigation (50), as well as drugs targeting integrin α4β7 and its vascular ligand, mucosal vascular addressin cell adhesion molecule 1 (MAdCAM-1) (46, 51).

Several drugs exert their therapeutic effect by influencing neutrophil chemotaxis and activation (52). In line with the suppression of immune cell trafficking molecules, we found that regulation of the actin cytoskeleton organization is crucial for maintaining barrier function and facilitating immune cell recruitment, and that CU104 stabilizes the cytoskeleton through the ERM pathway. The actin cytoskeleton, a key component of IECs, is essential for maintaining barrier integrity by regulating cell-cell adhesion and junctional complexes (34, 53). Rho-associated kinase ROCK1 suppresses the migration of inflammatory cells, such as neutrophils and macrophages (53). It plays a pathological role in vascular endothelial cells and M1 macrophages within the intestine, inhibiting the immune response by dampening IRF3 activity (54, 55). Due to its central role as a hub in immune cell trafficking and barrier function, ROCK1 might be a therapeutic target for controlling the activities of neutrophils and macrophages in different infectious and inflammatory conditions (53). Our findings suggest that regulating cytoskeleton dynamics might present an intriguing therapeutic strategy for alleviating barrier abnormalities and immune cell migration, suggesting specific mechanisms responsible for improving barrier function and reducing inflammation in colitis. Despite these findings, this study has several limitations. First, although the DNBS model and the DSS-triggered IL-10 KO model capture key pathological features relevant to IBD, the protocols employed here primarily reflect acute or subacute inflammation and therefore may not fully recapitulate the chronic, relapsing nature of human IBD. Second, while our data support the involvement of NF-κB/IRF signaling and cytoskeletal regulation (e.g., ROCK1/ERM-related pathways) in the protective effects of CU104, the precise direct molecular targets and cell-type–specific mechanisms remain to be determined. Third, we did not assess effects on additional intestinal compartments (e.g., mesenchymal cells), long-term outcomes, or microbiome-associated responses. Future studies using chronic/recurrent colitis paradigms (e.g., repeated DSS cycles), additional IBD models (e.g., T-cell transfer), and human-relevant systems (patient-derived organoids or ex vivo clinical samples) will be important to validate and extend these findings.

## Conclusions

This study demonstrated that CU104 effectively reduces CD-like colitis, regardless of IL-10 involvement, including Tregs. Furthermore, we identified key signaling pathways and molecular determinants by which CU104 effectively enhances vascular and intestinal barrier function, mitigates the recruitment, cytokine production, and activation of neutrophils by suppressing IRF and NF-κB, and protects against the collapse of ERM signaling by inflammation. These promising results highlight the efficacy of CU104 as a novel therapeutic agent for IBD.

## Data Availability

The original contributions presented in the study are included in the article/**Supplementary Material**. Further inquiries can be directed to the corresponding author.
