# Supplementary material for: CU104, a novel barrier function enhancer, improves colitis via modulation of barrier function and immune cell recruitment
Source: Front Immunol. 2026 Mar 10;17:1767762. doi: 10.3389/fimmu.2026.1767762 (PMC13010088; doi:10.3389/fimmu.2026.1767762)
Supplement: Supplementary file 1 [file DataSheet1.pdf]

CU104, a novel barrier function enhancer, improves colitis via modulation of barrier function and immune cell recruitment via modulation of actin dynamics

I Seul Park [1], Ji Hyung Kim[1], Dongyeop Kim[2], Ye Won Kim[1], YooJin Shin[1], Ki Beom Kim[1], Haiying Zhang[3], Tae Il Kim[1], Seung Won Kim[1,4†], Young-Guen Kwon[2,3], Jae Hee Cheon[1,4]

[1]Department of Internal Medicine and Institute of Gastroenterology, Graduate School of Medical Science, Brain Korea 21 Project, Yonsei University College of Medicine, Seoul, Korea

[2]Department of Biochemistry, College of Life Science and Biotechnology, Yonsei University, Seoul, Korea

[3]Curacle Co. Ltd., Seoul, Korea

[4]Severance Biomedical Science Institute, Yonsei University College of Medicine, Seoul, Korea

Table S1. Mouse primer sequences for qRT-PCR

Gene	Forward primer sequence (5' → 3')	Reverse primer sequence (5' → 3')
Tnfa	CAAAGGGAGAGTGGTCAGGT	ATTGCACCTCAGGGAAGAGT
Il1b	GCAACTGTTCCTGAACTCAACT	ATCTTTTGGGGTCCGTCAACT
Ifng	ATGAACGCTACACACTGCATC	CCATCCTTTTGCCAGTTCCTC
Nos2	GGCAGCCTGTGAGACCTTTG	GCATTGGAAGTGAAGCGTTTC
Cdh1	CTCCAGTCATAGGGAGCTGTC	TCTTCTGAGACCTGGGTACAC
Cldn1	CACTCCCAGACTCCACCACC	CGATCCATCCCAGAGAAGCC
Cldn2	CAACTGGTGGGCTACATCCTA	CCCTTGGAAAAGCCAACCG
Cxcl2	AACATCCAGAGCTTGAGTGTGA	TTCAGGGTCAAGGCAAACTT
Cxcl9	GGAGTTCGAGGAACCCTAGTG	GGGATTTGTAGTGGATCGTGC
Cxcr2	ATGCCCTCTATTCTGCCAGAT	GTGCTCCGGTTGTATAAGATGAC
Il10	CCCATTCCTCGTCACGATCTC	TCAGACTGGTTTGGGATAGGTTT
Il10rb	ACCTGCTTTCCCCAAAACGAA	TGAGAGAAGTCGCACTGAGTC
Il22	ATGAGTTTTTCCCTTATGGGGAC	GCTGGAAGTTGGACACCTCAA
Vcam1	TTGGGAGCCTCAACGGTACT	GCAATCGTTTTGTATTCAGGGGA
Icam1	GTGATGCTCAGGTATCCATCCA	CACAGTTCTCAAAGCACAGCG
Mmp7	CTTACCTCGGATCGTAGTGGA	CCCCAACTAACCCTCTTGAAGT
Mmp9	GCAGAGGCATACTTGTACCG	TGATGTTATGATGGTCCCACTTG
Mmp12	CTGCTCCCATGAATGACAGTG	AGTTGCTTCTAGCCCAAAGAAC
Ocln	CTCTCAGCCAGCGTACTCTT	CTCCATAGCCACCTCCGTAG
Kdr	ACCAGAAGTAAAAGTGATCCCAGA	TCCACCAAAAGATGGAGATAATTT
Actb	AGTGTGACGTTGACATCCGT	TGCTAGGAGCCAGAGCAGTA

Table S2. Human primer sequences for qRT-PCR

Gene	Forward primer sequence (5' → 3')	Reverse primer sequence (5' → 3')
CD11B	GGAACGCCATTGTCTGCTTTCG	ATGCTGAGGTCATCCTGGCAGA
TNFA	ATCTTCTCGAACCCCGAGTG	GGGTTTGCTACAACATGGGC
IL1B	AGCTACGAATCTCCGACCAC	CGTTATCCCATGTGTCGAAGAA
IFNG	TCGGTAACTGACTTGAATGTCCA	TCGCTTCCCTGTTTTAGCTGC
IL22	TGAATAACTAACCCCCTTTCCCTG	TGGCTTCCCATCTTCCTTTTG
IL8	CTCTTGGCAGCCTTCCTGATT	TATGCACTGACATCTAAGTTCTTTAG CA
ACTB	CTCTTCCAGCCTTCCTTCCTG	CAGCACTGTGTTGGCGTACAG

Table S3. Body weight (%) data used to generate Figure 1C

	Day 0	Day 1	Day 2	Day 3	Day 4	Day 5
**Control**	100.0	115.7 ± 0.8	119.8 ± 1.9	120.3 ± 1.2	123.4 ± 2.3	121.2 ± 2.3
**DNBS+Veh**	100.0	94.0 ± 0.5	89.6 ± 1.5	89.0 ± 3.6	94.4 ± 4.7	100.8 ± 4.0
**DNBS+CU104**	100.0	96.9 ± 0.7	95.5 ± 2.2	101.0 ± 2.8	106.7 ± 2.8	112.1 ± 1.7
**DNBS+Tofa**	100.0	94.7 ± 0.3	94.1 ± 2.0	95.0 ± 3.3	99.9 ± 4.1	103.2 ± 5.6

Data are presented as mean ± SEM (n = 4–10 mice per group).

Table S4. DAI score data used to generate Figure 1D

	Day 0	Day 1	Day 2	Day 3	Day 4	Day 5
**Control**	0.0	0.0 ± 0.0	0.0 ± 0.0	0.1 ± 0.1	0.0 ± 0.0	0.0 ± 0.0
**DNBS+Veh**	0.0	5.1 ± 0.3	6.1 ± 0.6	6.4 ± 0.9	5.0 ± 0.9	3.4 ± 0.5
**DNBS+CU104**	0.0	3.4 ± 0.3	4.0 ± 0.4	2.8 ± 0.5	1.8 ± 0.6	0.9 ± 0.1
**DNBS+Tofa**	0.0	4.4 ± 0.2	4.5 ± 0.4	4.1 ± 1.0	3.2 ± 0.8	2.3 ± 1.1

Data are presented as mean ± SEM (n = 4−10 mice per group).

Table S5. Body weight (%) data used to generate Figure 2B

	Day 0	Day 1	Day 2	Day 3	Day 4
**Control**	100.0	118.3 ± 1.5	119.7 ± 1.9	120.1 ± 1.9	122.7 ± 1.7
**DNBS+Veh**	100.0	96.4 ± 0.7	92.4 ± 1.4	94.1 ± 2.5	96.6 ± 2.9
**DNBS+CU104**	100.0	99.7 ± 1.3	104.1 ± 3.6	106.4 ± 4.9	114.6 ± 3.3
**DNBS+Oza**	100.0	97.8 ± 1.4	102.4 ± 2.9	106.8 ± 5.1	109.0 ± 6.0

Data are presented as mean ± SEM (n = 6–11 mice per group).

Table S6. DAI score used to generate Figure 2B

	Day 0	Day 1	Day 2	Day 3	Day 4
**Control**	0.0	0.1 ± 0.1	0.1 ± 0.1	0.0 ± 0.0	0.1 ± 0.1
**DNBS+Veh**	0.0	4.6 ± 0.4	6.1 ± 0.7	5.5 ± 0.6	4.3 ± 0.8
**DNBS+CU104**	0.0	3.1 ± 0.3	3.2 ± 0.6	2.8 ± 0.8	1.2 ± 0.1
**DNBS+Oza**	0.0	3.7 ± 0.4	3.3 ± 0.6	3.0 ± 0.9	2.6 ± 0.9

Data are presented as mean ± SEM (n = 6−11 mice per group).

Table S7. Body weight (%) data used to generate Figure 4B

	Day 0	Day 1	Day 2	Day 3	Day 4	Day 5	Day 6	Day 7	Day 8	Day 9
**Water**	100.0	99.9 ± 1.2	100.4 ± 0.5	99.7 ± 0.3	100.1 ± 0.3	101.4 ± 0.8	102.2 ± 0.8	102.3 ± 0.8	102.3 ± 0.6	102.8 ± 0.6
**DSS+Veh**	100.0	100.1 ± 0.8	100.9 ± 1.1	100.1 ± 0.4	97.6 ± 0.9	98.7 ± 0.7	96.5 ± 1.0	93.5 ± 1.6	88.2 ± 2.4	84.4 ± 3.2
**DSS+CU104 (10 mg/kg)**	100.0	98.3 ± 1.0	99.6 ± 0.5	97.7 ± 1.4	95.8 ± 1.6	97.6 ± 1.3	96.8 ± 1.2	96.3 ± 1.0	93.4 ± 0.9	89.7 ± 1.7
**DSS+CU104 (20 mg/kg)**	100.0	100.4 ± 1.7	99.5 ± 2.3	98.7 ± 2.7	96.8 ± 2.6	98.6 ± 2.1	98.4 ± 1.9	98.2 ± 2.1	96.8 ± 2.3	92.9 ± 2.5
**DSS+5-ASA**	100.0	100.3 ± 0.4	100.0 ± 0.5	100.5 ± 0.9	97.6 ± 0.9	98.9 ± 0.6	97.9 ± 1.0	96.0 ± 1.9	92.0 ± 2.5	88.7 ± 3.3

Data are presented as mean ± SEM (n = 10 mice per group).

Table S8. DAI score data used to generate Figure 4C

	Day 0	Day 1	Day 2	Day 3	Day 4	Day 5	Day 6	Day 7	Day 8	Day 9
**Water**	0.0	0.1 ± 0.1	0.0 ± 0.0	0.2 ± 0.1	0.1 ± 0.0	0.1 ± 0.1	0.0 ± 0.0	0.0 ± 0.0	0.0 ± 0.0	0.0 ± 0.0
**DSS+Veh**	0.0	0.2 ± 0.1	0.1 ± 0.1	0.1 ± 0.1	0.2 ± 0.1	0.3 ± 0.1	0.9 ± 0.2	1.6 ± 0.3	2.3 ± 0.3	2.7 ± 0.3
**DSS+CU104 (10 mg/kg)**	0.0	0.3 ± 0.1	0.2 ± 0.1	0.3 ± 0.1	0.4 ± 0.1	0.4 ± 0.3	0.6 ± 0.1	0.9 ± 0.1	1.2 ± 0.1	1.3 ± 0.1
**DSS+CU104 (20 mg/kg)**	0.0	0.2 ± 0.1	0.3 ± 0.1	0.2 ± 0.1	0.3 ± 0.1	0.3 ± 0.1	0.6 ± 0.1	0.6 ± 0.1	0.6 ± 0.2	0.9 ± 0.2
**DSS+5-ASA**	0.0	0.3 ± 0.1	0.2 ± 0.1	0.3 ± 0.2	0.4 ± 0.1	0.4 ± 0.1	0.7 ± 0.2	0.6 ± 0.2	1.0 ± 0.2	1.4 ± 0.3

Data are presented as mean ± SEM (n = 10 mice per group).
